# Application of unilateral external fixation by the “joystick technique” in the treatment of pediatric tibia shaft fractures: technical note

**DOI:** 10.1186/s13018-021-02625-w

**Published:** 2021-08-12

**Authors:** Xinhui Wang, Zhe Zhang, Xizhi Hou, Bao Wang, Yongdong Li, Tao Zhang

**Affiliations:** 1 Emergency, Trauma Center, The Third Hospital of Hebei Medical University, No.139 Ziqiang Road of Qiao’xi District, Shijiazhuang, 050000 Hebei China; 2 Monitoring and Evaluation Office, Health Guidance Center of Hebei Provincial Health Commission, Shijiazhuang, China; 3 Three Wards of Traumatology Orthopedics, The Third Hospital of Shijiazhuang City, Shijiazhuang, Hebei Province China

**Keywords:** Pediatric tibia shaft fracture, Unilateral external fixation, Joystick technique

## Abstract

**Background:**

The aims of current study were to present the clinical outcomes in patients with pediatric tibia shaft fractures who were treated with unilateral external fixation combined with joystick for fracture reduction and describe the details of our technique.

**Methods:**

We retrospectively analyzed the patients with pediatric tibia shaft fractures who were treated with unilateral external fixation combined with joystick for fracture reduction between July 2018 and March 2020. The clinical outcomes were evaluated.

**Results:**

A total of 23 patients were included in the current study with the average age of 8.0 years (ranged 4–14 years). The average duration of hospital and follow-up were 5.9 days (ranged 4–8 days) and 17.4 months (ranged 8–27 months), respectively. At postoperative 3 days, the visual analog scale (VAS) score was 3.1 ± 1.43, which was significantly lower than the preoperative score of 7.3 ± 1.5. Of these, 2 cases showed redness and swelling of pin-tract and exudation at postoperative 1 month, who improved after oral antibiotics without causing fixation failure. The average time to full weight-bearing without crutches was 5.1 weeks (ranged 3–8 weeks). All patients achieved fracture healing and good functional recovery. No complications including fixation failure, reoperation, epiphyseal injury occurred, infection around implants, vessel damage, nerve damage, and limitation of joint movement were observed. The Johner-Wruh scores showed that 21 cases (91.3%) were “excellent” and 2 cases (8.7%) were “good.”

**Conclusions:**

This procedure had advantages of simple operation, minimum trauma, early recovery of lower limb function, and no risk of complications. It may provide a new choice for children with tibia shaft fractures who require surgical treatment.

## Background

Tibia fracture is a common injury in children accounting for 15% of children’s fractures, and 40% of tibia fractures are located in the middle of the tibia [1–3]. Since pediatric fractures with characteristics of faster healing and strong shaping ability, cast external fixation was treated for was tibia shaft fractures with insignificant displacement, which achieved satisfactory outcomes, especially for greenstick fractures [4]. However, for significantly displaced tibia fractures, surgical treatment should be considered to avoid sequelae caused by shortening, rotation, or severe force line changes. Recently, the surgical approaches for treating tibia shift fractures include external fixation, titanium elastic nail (TEN), and open reduction and internal fixation [5–7]. Despite the development of these surgery methods, open reduction and internal fixation has several disadvantages including large trauma, destruction of blood supply, and increases the risk of bone nonunion and infection [5, 8]. At the same time, the inevitable surgery to remove internal fixation will cause secondary injury to the child. Therefore, the minimally invasive surgical treatment of pediatric tibia shaft fractures is urgent.

TEN has been first reported in 1988 by Ligier et al. [9], which was quickly accepted and promoted for use in pediatric tibia shaft fractures. Previous studies have demonstrated that TEN is beneficial for the treatment of pediatric tibia shaft fractures [8, 10, 11]. In practical application, TEN is most suitable for transverse or short oblique fractures. However, its fixation strength is reduced for other fracture types such as long oblique or spiral fractures, and it needs to be combined with external fixation with a brace, thereby influencing early functional exercise [8, 12]. Additionally, TEN can cause secondary surgery to remove internal fixation as well [7]. With regard to external fixation, it is deemed as a primary and effective treatment for pediatric tibia shaft fractures despite the complications [5, 13]. But there are still few studies on the use of external fixation to treat tibia shaft fractures in children, and a mature treatment system has not been formed.

Translational orthopedics is that new orthopedic technique which is efficiently transformed from the scientist to the clinician [14]. In this study, we tried to apply a unilateral external fixator combined with joystick for fracture reduction in the treatment of pediatric tibia shaft fractures. In order to accelerate understanding the clinical application, we described the details of this technique and explored the clinical outcomes.

## Methods

### Patients

Children with tibia shaft fractures who were admitted to the Trauma Emergency Center of the Third Hospital of Hebei Medical University between July 2018 and March 2020 were retrospectively analyzed in this study. Inclusion criteria were patients who (1) were aged from 4 to 14 years, (2) were closed or Gustilo I type tibia shaft fracture, and (3) had obvious displacement or unstable fracture and poor closed reduction. Exclusion criteria were patients who had (1) fractures at other sites, (2) Gustilo II or Gustilo III type tibia shaft open fracture, (3) closed fracture with nerve and blood vessel damage, and (4) other severe disabilities or medical diseases.

### Surgery procedure

External fixation was performed in all patients under general anesthesia. After successful anesthesia, the patient was placed in the supine position on the operating beds. In general, half-pin with a diameter of 4 mm was used to fix the tibia shaft fractures in children and 5/6 mm diameter for individual patients with larger body weight.

**Fig. 1:**
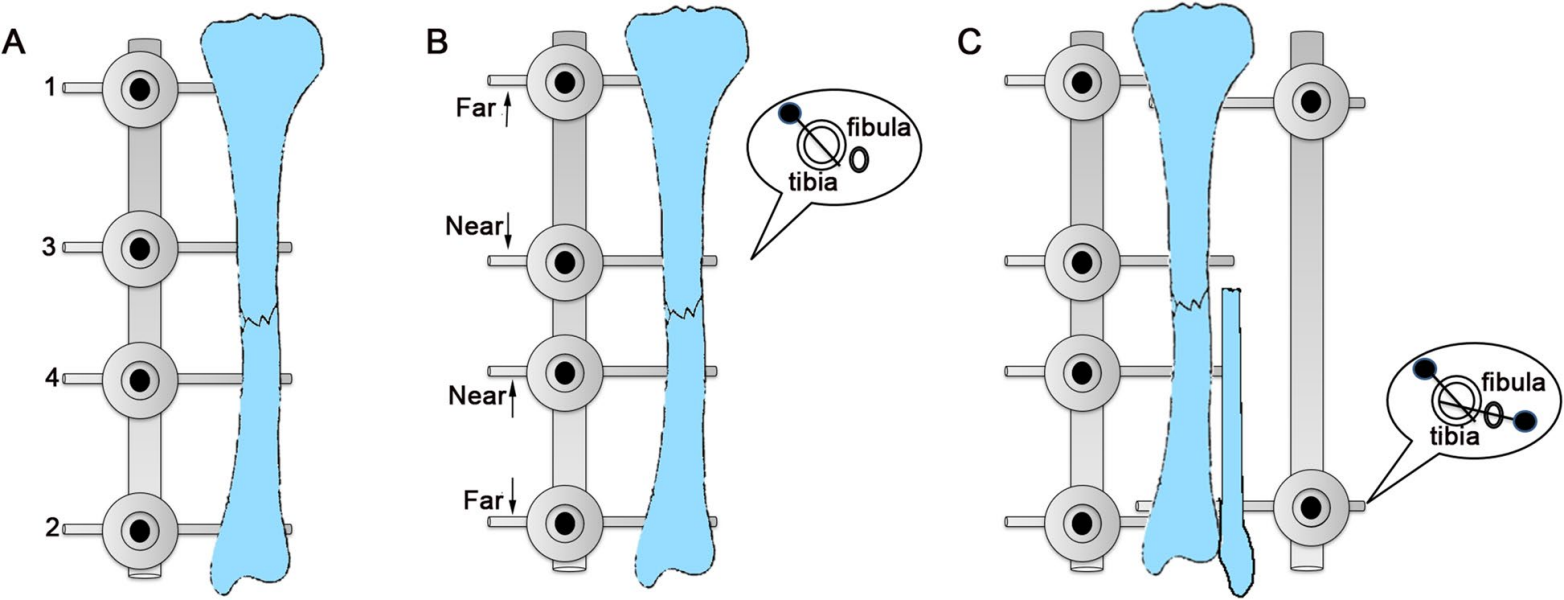
The fixation skills of pediatric tibia shaft fractures. **A** The sequence of placement of half-pins in turn at the nearest (1), farthest (2), and close to the fracture (3, 4). **B** The selection of the position followed the “near-near, far-far principle,” and 4 half-pins were inserted from the inner surface of the tibia perpendicular to the bone surface. The outside fixator needed 2 half-pins. **C** The proximal end was located below the level of tibia tubercle, and the distal end was driven through the fibula into the tibia

To reduce soft tissue irritation, simple fractures should be fixed with 4 half-pins first. After the fracture was reduced and fixed, if necessary, additional half-pins were added to each fracture segment to increase the fixation strength [15]. The half-pins were fixed in turn at the nearest, farthest, and close to the fracture (Fig. 1A). All 4 half-pins were located on the anterior medial surface of the tibia [2]. In order to increase the holding force of the external fixation frame, the selection of the position of the half-pins followed the “near-near, far-far principle.” That is, the most farthest and nearest half-pins should be as far away from the fracture as possible, and the middle half-pins should be as close as possible to the fracture [15] (Fig. 1B).

Especially for patients with older age, another unilateral external fixator was added to the outside. The outside fixator needed 2 half-pins. The proximal end was located below the level of tibia tubercle, and the distal end was driven through the fibula into the tibia. The connecting rod was used to connect the lateral half-pin to increase stability (Fig. 1C).

After inserting the distal and proximal half-pins, traction reduction was performed to restore the tibia rotation and the length of the tibia. The distal and proximal nail caps were tightened to firmly fix the half-pins and connecting rods. The intraoperative C-arm fluoroscopy of the fracture in the anteroposterior and lateral position was used to detect whether the fracture length is restored. On the premise of confirming the length recovery, the dislocation of the fracture was detected.

**Fig. 2:**
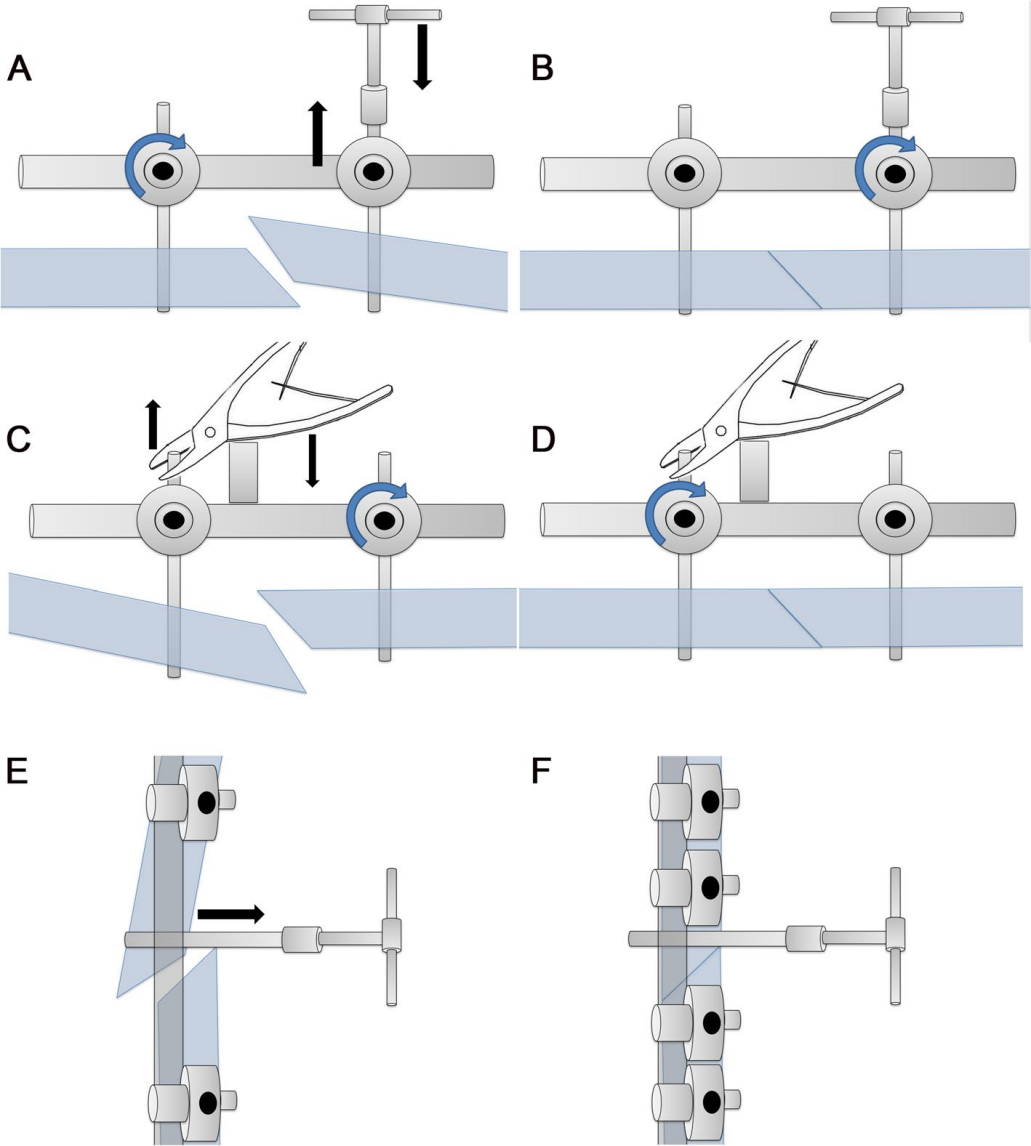
Application of joystick technique in pediatric tibia shaft fractures. **A**–**D** Two half-pins were inserted into close to the fracture, and then the pressing technique or leverage method was adopted according to the displacement. **E**, **F** Inserting a half-pin as joystick into the anterior tibia crest near the fracture was used for anteroposterior displacement. After closed and reduction of the fracture, half-pins were placed on each side of the fracture to fix

Since the external fixator is located on the inner side of the tibia, if there was only mediolateral displacement of the fracture but no anteroposterior displacement, two half-pins were inserted into close to the fracture first. Subsequently, according to the displacement, the pressing technique or leverage method was adopted. After pressing or lifting the fracture, satisfactory reduction was obtained by tightening the nail cap (Fig. 2A–D). If there was anteroposterior displacement, inserting a half-pin as joystick into the anterior tibia crest near the fracture was recommended. After closed and reduction of the fracture under fluoroscopy, half-pins were placed on each side of the fracture to fix, and satisfactory reduction was also obtained (Fig. 2E, F).

### Postoperative management

Postoperatively, the affected limb was elevated and intravenous antibiotics were used for 48 h of anti-infective treatment. On the second day after the operation, the patients were instructed to perform non-weight-bearing exercises of the knee and ankle joints on the premise that the pain was tolerable after the pain subsided. One week after the operation, the patients were instructed to move on crutches after reduction of the swelling of the affected limb. Beginning 2–3 weeks after the operation, the patients were instructed to exercise the weight-bearing function of the affected limb using a weight scale under the protection of crutches.

Bone union was evaluated by the X-ray results of reexamination after surgery according to the previous study [12]. Briefly, when an adequate bridging callus was observed on a radiograph, the patients were instructed to walk with full weight-bearing gradually. When patients can take off the crutches to walk with no pain, we believed that the patient has reached clinical healing. After clinical healing was achieved, half-pins were gradually removed in 2–3 times until they were completely removed. The interval time for each removal of external fixator was about 1 month.

### Clinical outcomes

Visual analog scale (VAS) was to assess the pain at preoperation and postoperative 3 days. During the follow-up, the fracture healing, complications, and functional recovery were recorded. At the last follow-up, the patient’s recovery was evaluated according to the Johner-Wruh tibia fracture curative effect evaluation system [16] (Table 1).

**Table 1:** Johner-Wruh scoring system

	Excellent	Good	Medium	Poor
Infection/non-healing	No	No	No	Yes
Neurovascular injury	No	Mild	Moderate	Severe
Varus and valgus deformities	No	2°–5°	6°–10°	> 10°
Anterior and posterior bending deformities	0°–5°	6°–10°	11°–20°	> 20°
Rotation deformity	0°–5°	6°–10°	11°–20°	> 20°
Shortening deformity	0°–5°	6°–10°	11°–20°	> 20°
Knee range of motion	Unlimited	> 80%	> 75%	< 75%
Ankle range of motion	Unlimited	> 75%	> 50%	< 50%
Pain	No	Occasional	Moderate	Severe
Gait	Normal	Normal	Mild limp	Obvious limp
Daily activities	Unrestricted	Restricted	Severely restricted	Unable to take care of yourself

### Statistical analysis

Data were analyzed using SPSS 19.0 software (IBM Corp., Chicago IL, USA). The measurement data were compared using *t* test, while the enumeration data using chi-square test. *P* < 0.05 was considered to the statistically significant difference.

## Results

A total of 23 patients were included in the current study with the average age of 8.0 years (ranged 4–14 years). The average hospital duration and follow-up were 5.9 days (ranged 4–8 days) and 17.4 months (8–27 months), respectively. There were 14 males and 9 females, and there were 12 cases on the left side and 11 cases on the right side. The injury was caused as a result of traffic accidents in 9 cases (39.1%), sprain in 9 cases (39.1%), heavy objects in 3 cases (13.0%), and other causes in 2 cases (8.7%) (Table 2).

**Table 2:** Patient characteristics

Case	Age (years)	Gender	Fracture side	Injury causes	Duration of hospital (day)	Weight-bearing (day)	Complications	Johner-Wruh score
1	4	Male	Left	Heavy objects	4	3	No	Excellent
2	13	Male	Right	Traffic accident	8	6	No	Excellent
3	5	Male	Left	Traffic accident	7	4	No	Excellent
4	9	Male	Left	Heavy objects	6	8	Pin tract infection	Good
5	12	Male	Right	Sprain	4	5	No	Excellent
6	4	Male	Left	Traffic accident	5	6	No	Excellent
7	7	Female	Right	Traffic accident	6	4	No	Excellent
8	13	Male	Left	Traffic accident	5	5	Pin tract infection	Excellent
9	6	Female	Left	Other	6	4	No	Excellent
10	11	Male	Right	Sprain	4	3	No	Excellent
11	8	Male	Left	Sprain	8	6	No	Excellent
12	7	Female	Right	Sprain	7	5	No	Excellent
13	4	Female	Right	Other	5	4	No	Excellent
14	5	Male	Left	Traffic accident	6	4	No	Excellent
15	14	Male	Right	Traffic accident	4	5	No	Excellent
16	10	Female	Left	Sprain	4	7	No	Good
17	8	Female	Right	Sprain	5	5	No	Excellent
18	3	Male	Left	Heavy objects	7	5	No	Excellent
19	7	Female	Right	Sprain	7	4	No	Excellent
20	10	Female	Right	Traffic accident	8	6	No	Excellent
21	9	Male	Left	Sprain	5	5	No	Excellent
22	8	Female	Right	Traffic accident	7	7	No	Excellent
23	7	Male	Left	Sprain	8	6	No	Excellent

**Fig. 3:**
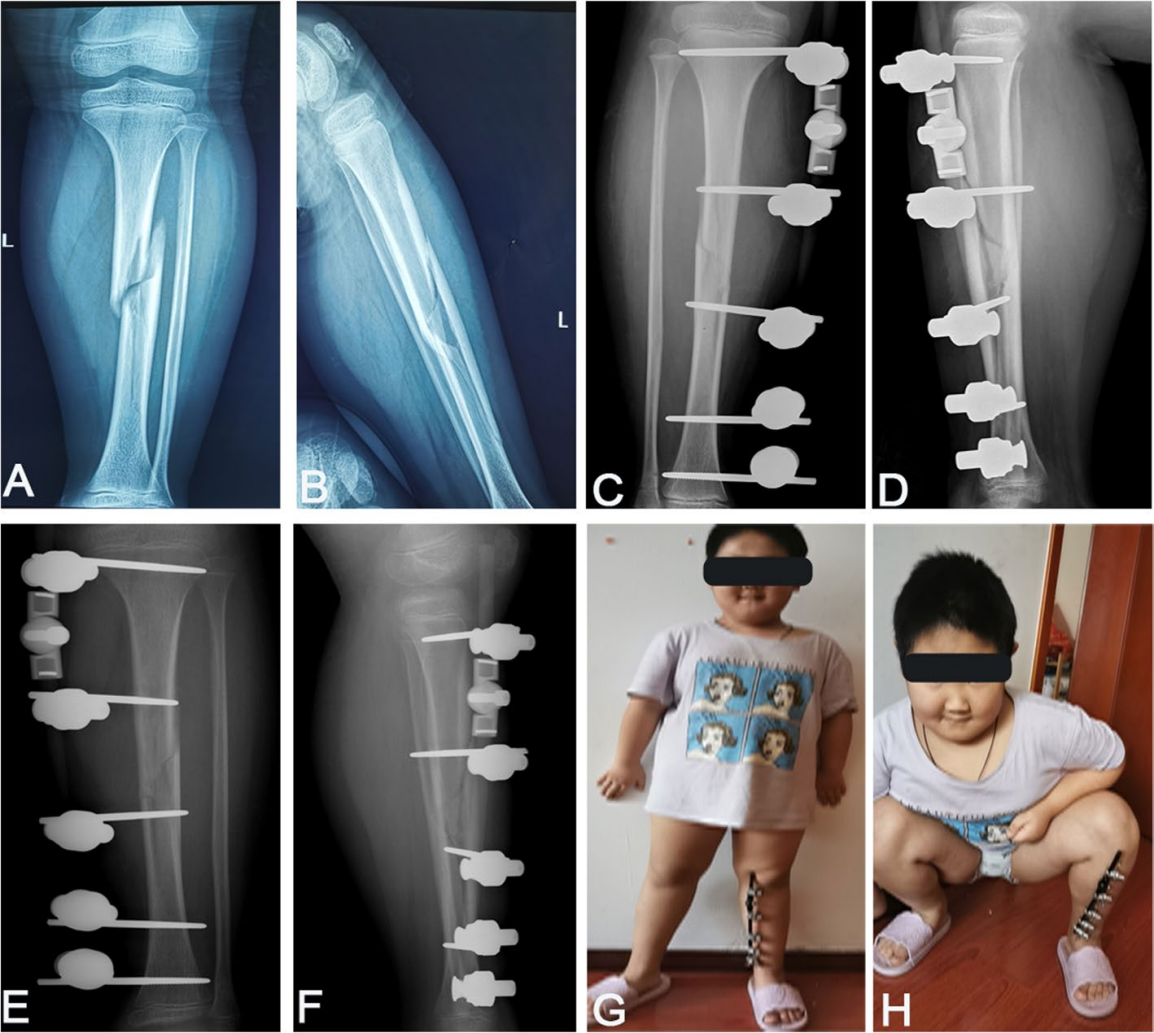
Representative cases with tibia shaft fractures at preoperation and postoperation. **A**, **B** X-ray images of tibia shaft fractures of an 8-year-old boy. **C**, **D** X-ray images at postoperative immediate showed good reduction. **E**, **F** X-ray images at postoperative 1 month. **G**, **H** At 5 weeks postoperatively, the patient walked without crutches and recovered well

**Fig. 4:**
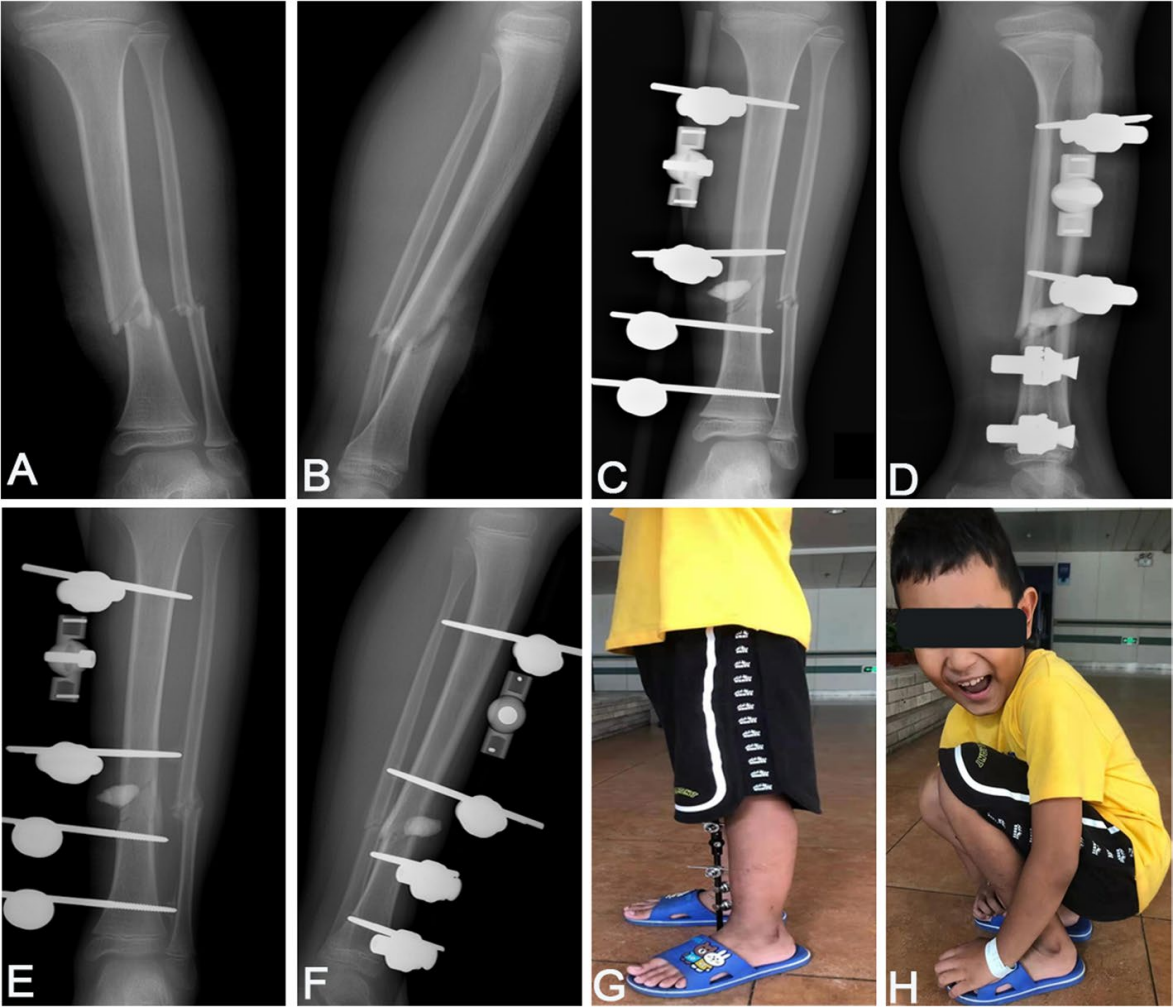
Representative cases with Gustilo type I open tibia-fibula shaft fractures at preoperation and postoperation. **A**, **B** X-ray images of a 7-year-old boy at preoperation. **C**, **D** X-ray images at postoperative immediate showed good reduction and use of local antibiotic bone cement to prevent infection. **E**, **F** X-ray images at postoperative 1 month. **G**, **H** At 4 weeks postoperatively, the patient walked without crutches and recovered well

At postoperative 3 days, the average VAS score of patients was 3.1 ± 1.43, which was significantly lower than the preoperative score of 7.3 ± 1.5 (Table 3). Of these, 2 cases showed redness and swelling of pin-tract and exudation at postoperative 1 month. Fortunately, these conditions were controlled through oral antibiotic treatment and pin tract care. The average time to full weight-bearing without crutches was 5.1 weeks (ranged 3–8 weeks). The maintenance period of the external fixator was 13.5 weeks (ranged 10–17 weeks). All patients achieved fracture healing, and no complications were observed including fixation failure, reoperation, epiphyseal injury occurred, infection around implants, vessel damage, nerve damage, and limitation of joint movement (Figs. 3 and 4).

**Table 3:** Comparison of VAS score between preoperation and postoperation

Preoperation (mean ± SD)	Postoperation (mean ± SD)	*t* value	*P* value
7.3 ± 1.5	3.1 ± 1.4	9.6	< 0.05

During follow-up of 12 months, the patients showed good functional recovery, and none of them showed obvious unequal length of lower limbs and claudication. The Johner-Wruh scores at the end of 12 months were “excellent” for 21 cases (91.3%) and “good” for 2 cases (8.7%). The advantages and limitations of this study were shown in Table 4.

**Table 4:** The advantages and limitations in the study

Advantages	Limitations
A new technology attempt	Small sample
Minimal-invasive approach	Single arm retrospective study
Early recovery of lower limb function	Single center study
Simple operation	–
Avoiding second operation	–

## Discussion

External fixation is a common surgical technique for tibia-fibula fractures, especially for open tibia-fibula fractures. In general, external fixation is utilized as a temporary fixation to restore the length and force of the tibia and fibula, and then changed to internal fixation treatment, which can often achieve satisfactory outcomes [17, 18]. With the advancements of external fixation technology and equipment, it is deemed as ultimate treatment for tibia-fibula fractures, and even applied to the treatment of closed fractures, which achieves good therapeutic effects [19, 20]. Combined with the children characteristics of fast healing and strong shaping ability, the external fixation has superiority of small trauma and no secondary surgery, which is deemed as a reasonable treatment option [13, 21]. However, there are still few studies on the use of external fixation to treat tibia shaft fractures in children. A previous study has reported that unilateral external fixator combined with limited open reduction was used to treat in pediatric tibia fractures, and it found that the therapeutic effect was satisfactory [22]. Joystick technique refers to the use of metal bone pin into the fracture, followed by reaching the purpose of reduction. Since joystick technique can achieve closed reduction of fractures that originally required open reduction, it is becoming an emerging method in the management of fractures [23–25]. In the current study, unilateral external fixator combined with joystick for fracture reduction was used for pediatric tibia shaft fractures, which obtained benefit effects.

Average hospital duration in the current study was 5.9 days, which was lined with previous study using monolateral external fixation combined with open reduction [22]. In the aspects of weight-bearing, we found that the average time was keeping with the abovementioned previous study [22], however superior to TEN type of surgical treatment [11, 26]. In our series, full fracture healing and no complications were observed in all patients, which differ from the outcomes using external fixation in the Gordon et al. [27] report. However, a recent study has used the hybrid external fixation by the joystick method in the bicondylar tibia plateau fractures, and then found the full fracture healing in all cases [28]. Importantly, certain matters should be paid attention during the surgery process, which may be closely associated with the therapeutic effect. Briefly, reduction of the local temperature during the drilling or half-insertion of the needle can effectively avoid osteonecrosis induced by excessive temperature. The sharpness of the drill is important as well. Additionally, ensuring that each half-pin was inserted into bicortex in our study, which has been proved in the previous study [29].

As we have known, the challenges faced in external fixation are mainly the fixation strength and pin-tract infection in the treatment of pediatric tibia shaft fractures [2]. Since external fixator cannot achieve the same nail density as the locking bone plate, how to obtain the maximum fixation strength under the premise of placing the limited number of screws is the key to the success of the treatment [15]. A previous study has used unilateral external fixators to treat the 29 children with tibia shaft fractures, of which 4 patients (13%) experienced loss of reduction [27]. Additionally, a retrospective study conducted by Parameswaran et al. [30] has showed that 11.2% of external fixation patients had pin tract infection. Furthermore, previous studies have documented the soft-tissue trauma as a risk factor for infection rate [31, 32]. In the current study, a variety of methods were used to minimize the stimulation of the soft tissues. First, half-pins were inserted from the inner surface of the tibia perpendicular to the bone surface. Second, No. 11 (sharp blade) or No. 15 (gun blade) was used to pre-cut and then half-pins were inserted. In addition, since the fracture was displaced, the distal and proximal half-pins were firstly inserted to restore the relative position of the fracture and soft tissue, and then the half-pins were placed adjacent to the fracture. After following the standard nail placement technique and reasonable care, only 2 patients (8.7%) had a slight pin-tract infection, who improved after oral antibiotics without causing fixation failure.

In children, fractures heal quickly, but new calluses are softer [33]. A previous study has revealed that weight-bearing may cause deformity or re-fracture in the lower limbs if the external fixation is completely removed after the fracture healing [2]. Additionally, Greene et al. [34] have found that local pressure stimulation can effectively promote bone formation and calcium accumulation, indicating gradually increasing the weight-bearing of the fracture site under the protection of external fixators may be an effective means to prevent re-fracture. In our study, the external fixation device is gradually removed for 2–3 times, when the patient recovered the function of the lower limbs and walked without crutches. Fortunately, none of the 23 patients in this study had fractures after removal of external fixation.

There were some limitations in the current study. Briefly, this study was single arm retrospective and conducted in a single center medical institution with a small sample.

## Conclusions

Unilateral external fixation combined with joystick for fracture reduction had advantages of simple operation, minimum trauma, early recovery of lower limb function, and no risk of complications. It may provide a new choice for children with tibia shaft fractures who require surgical treatment. However, a randomized controlled study will be conducted in the future to verify the efficacy.

## Data Availability

All data generated or analyzed during this study are included in this article.

## Abbreviations

TEN: Titanium elastic nail
VAS: Visual analog scale
